# Systemic immunity upon local oncolytic virotherapy armed with immunostimulatory genes may be supported by tumor-derived exosomes

**DOI:** 10.1016/j.omto.2021.02.007

**Published:** 2021-02-17

**Authors:** Alireza Labani-Motlagh, Sedigheh Naseri, Jessica Wenthe, Emma Eriksson, Angelica Loskog

**Affiliations:** 1 Department of Immunology, Genetics and Pathology, Science for Life Laboratory, Uppsala University, Uppsala, Sweden; 2 Lokon Pharma AB, 75185 Uppsala, Sweden

**Keywords:** CD40L, 4-1BBL, oncolytic adenovirus, exosomes, dendritic cells, costimulatory molecules, LOAd703

## Abstract

Immunostimulatory gene therapy utilizing oncolytic viruses (OVs) as gene vehicles is a promising immunotherapy for cancer. Since viruses are immunogenic, systemic delivery can be troublesome due to neutralizing antibodies. Nevertheless, local delivery by intratumoral injection seems to induce systemic immune reactions. In this study, we demonstrate a novel mechanism of action of armed OV therapy suggesting that exosomes released by tumor cells infected with armed OV may participate to activate the immune system and this may also support systemic immunity. Tumor cell-derived exosomes commonly exert immunosuppressive functions. We hypothesized that exosomes derived from OV-infected tumor cells may instead be immunostimulatory. Human melanoma cells were infected by OVs armed with costimulatory molecules CD40 ligand (CD40L) and 4-1BB ligand (4-1BBL). Exosomes were purified and investigated for the presence of CD40L/4-1BBL mRNA and protein, and for their capacity to stimulate immune responses. The results show that the exosomes cargo transgenes. The exosomes from CD40L/4-1BBL-expressing tumor cells, or the viruses themselves, could stimulate robust dendritic cell (DC) activation with an enhanced level of major histocompatibility complex (MHC) and costimulatory molecules. Hence, exosomes after OV infection can locally activate immune responses at the tumor site and encounter immune cells such as DCs.

## Graphical Abstract

**Figure:**
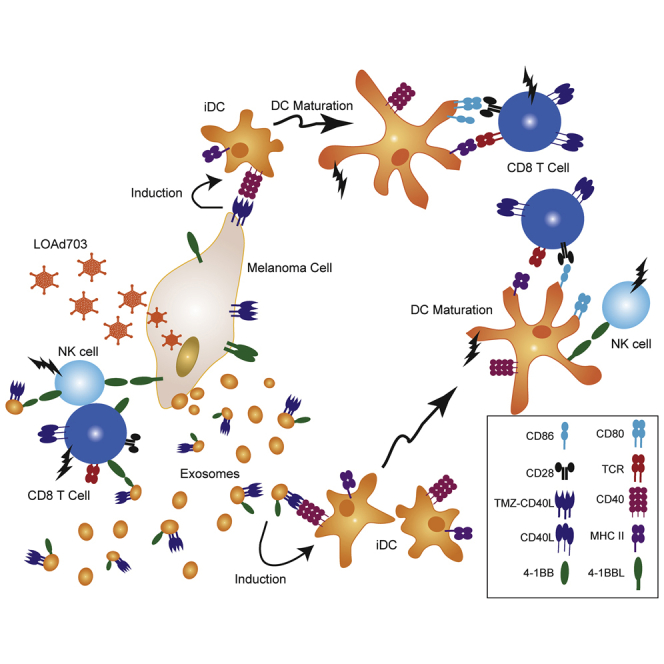

## Teaser

Tumor cells infected with armed oncolytic LOAd703 viruses release exosomes expressing two costimulatory transgenes CD40L and 4-1BBL, and such nanovesicles can activate dendritic cells *in vitro*. This behavior of tumor-derived exosomes is immunostimulatory rather than their immunosuppressive nature and can elicit antitumor immune responses.

## Introduction

The tumor microenvironment (TME) is heterogeneous and complex. As shown in several cancer types, the presence of tumor-infiltrating lymphocytes (TILs) in solid tumors is an attribution of better prognosis.^1^ However, antitumor responses are targeted by many immunosuppressive molecules and cancer-associated stroma.^2^ Although TILs have pivotal roles in clinical outcomes, their recruitment and survival in a solid tumor are not easily facilitated. Therefore, stimulation of TILs directly or with the help of antigen-presenting cells (APCs) such as dendritic cells (DCs) is required for elimination of tumor cells. Immunostimulatory gene therapy using oncolytic viruses (OVs) is an interesting option to inflame tumors and enhance TIL infiltration and functionality. OVs can infect cells in the TME but selectively replicate inside tumor cells, thereby destroying them in the process of oncolysis. Consequently, tumor-associated antigens can be presented by APCs to elicit antitumoral T cell responses. OVs armed with human immunostimulatory genes can further improve antitumor immunity by enhancing immune reactions in the TME.^3^ Such armed OVs are evaluated in clinical trials as a single treatment and in combination with checkpoint inhibition (CPI) antibodies.^4^^,^^5^ Our group previously designed LOAd oncolytic adenoviruses armed with immunostimulatory transgenes.^6^^,^^7^ For example, LOAd703 encodes TNFSF9 (4-1BB ligand;4-1BBL) and trimerized membrane-bound isoleucine zipper CD40 ligand (TMZ-CD40L derived from the CD40Lg gene), whereas LOAd700 encodes only TMZ-CD40L. As a member of the tumor necrosis factor (TNF) ligand family, 4-1BBL is endogenously expressed on activated APCs, T cells, and several carcinoma cell lines.^8^, ^9^, ^10^ Engagement of 4-1BBL with its receptor, 4-1BB, promotes costimulatory signal transduction in CD4^+^ and CD8^+^ T cells, leading to their activation and expansion of CD8^+^ T cells with enhanced IFN-γ production.^8^ Naturally found CD40L is expressed on activated B cells, T cells, platelets, monocytes, natural killer (NK) cells, basophils, and mast cells.^11^ Interaction between CD40L and its receptor culminates in DC maturation and activation.^11^ When LOAd viruses infect tumor cells, they can replicate, reprogram the cells to express the transgenes, and display them as membrane-bound proteins. Thus, they induce antitumor responses either directly or indirectly through DCs. We hypothesized that LOAd-infected tumor cells expressing the transgenes may pack them into the endosomal compartments and release them via a subtype of extracellular vesicles known as exosomes. Such transgene-expressing exosomes may participate to elicit the antitumor responses locally and likely at distant sites from the tumor.

Exosomes are cup-shaped nanovesicles 30–150 nm in diameter and originate from endosomal compartments in the cells during biogenesis.^12^ They carry proteins, lipids, nucleic acids, and metabolites.^13^ These extracellular vesicles participate in cell-cell communication and various functions such as antigen presentation, apoptosis, anti-apoptosis, angiogenesis, metastasis, coagulation, pro-inflammatory and anti-inflammatory responses, and immune activation or suppression.^14^ Furthermore, tumor-associated exosomes have shown immunosuppressive function because of their repressing molecules that reduce or hinder the function of immune cells upon encounter.^15^, ^16^, ^17^ In this study, we elucidate the capacity of exosomes from tumor cells infected by immunostimulating OVs to activate immune responses rather than suppressing them. We demonstrate herein a novel mechanism of action of how armed OVs can mediate immune activation via spreading their payload on tumor-derived exosomes. Our studies may also initiate investigations on how exosomes may participate in unwanted immunogenicity of any gene therapy vehicle in genetic diseases in which the immunostimulatory capacities of viruses are unwanted.

## Results

### Mel526 cells infected with LOAd700 and LOAd703 express their transgenes

**Figure 1:**
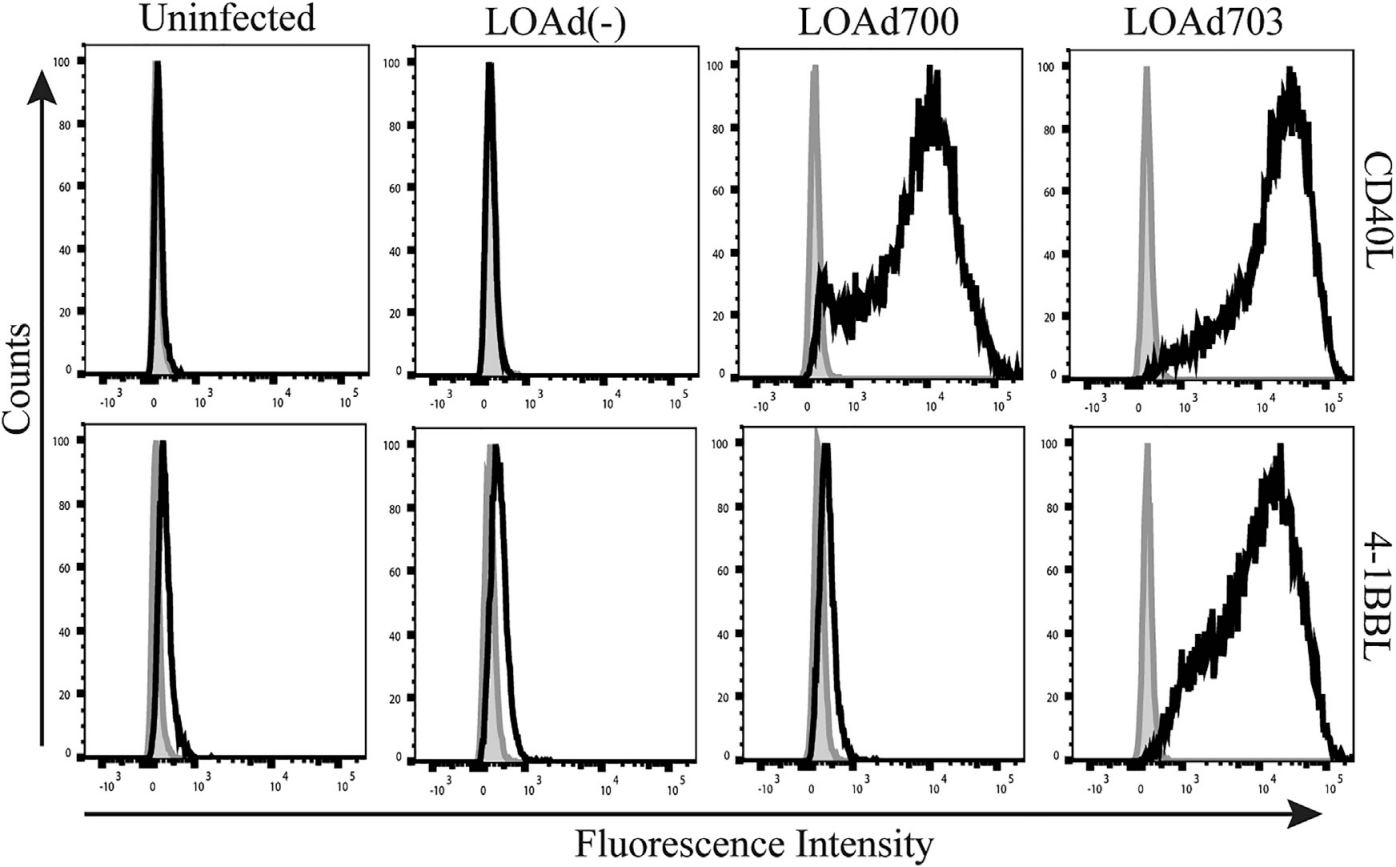
Expression of CD40L and 4-1BBL on Mel526 cells Mel526 cells infected with LOAd700 or LOAd703 express CD40L (upper histograms), while cells infected with LOAd703 also present 4-1BBL (lower histograms), as shown with flow cytometry at day 2 post-infection. Gray and black histograms represent isotype control and transgene staining, respectively.

The melanoma cell line Mel526 was used in this study. We infected the cells with LOAd700, LOAd703, LOAd(−) (10 multiplicities of infection [MOIs]/cell), or left them uninfected. We analyzed them for expression of the immunostimulatory transgene of the OVs. The gating strategy can be seen in Figure S1. Cells infected with LOAd700 or LOAd703 displayed CD40L expression compared with LOAd(−) and uninfected cells (Figure 1). In addition, only cells infected with LOAd703 highly expressed 4-1BBL in comparison to the low natural expression in these cells (Figure 1). These data indicate the successful transgene delivery into the tumor cells by LOAd viruses *in vitro* and their translation into proteins post-infection.

### LOAd700- and LOAd703-infected cells release exosomes displaying CD40L and 4-1BBL

**Figure 2:**
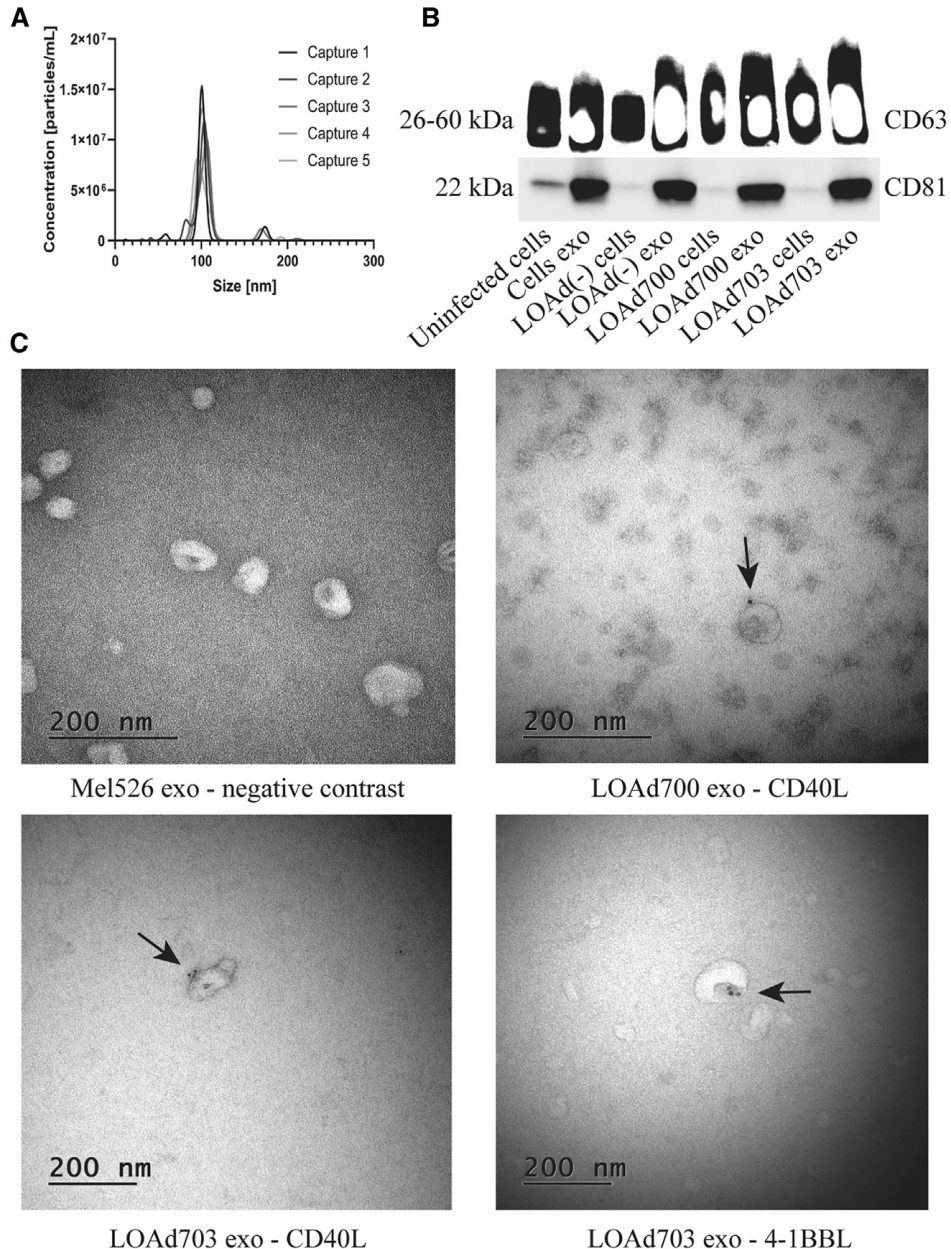
Characterization of exosomes (A) Nanoparticle tracking analysis of exosomes isolated from Mel 626 cells using NanoSight NS500. Captures 1–5 represent five captured videos for calculation of size and concentration of exosomes. (B) Exosomal markers (CD63 and CD81) were evaluated on cells (either infected or uninfected) and their exosomes using western blot. (C) Electron micrographs of isolated exosomes stained for CD40L or 4-1BBL. Arrows indicate positive staining. Cells exo, exosomes from uninfected cells; LOAd cells, cells infected with LOAd viruses; LOAd exo, exosomes released by LOAd-infected cells.

**Figure 3:**
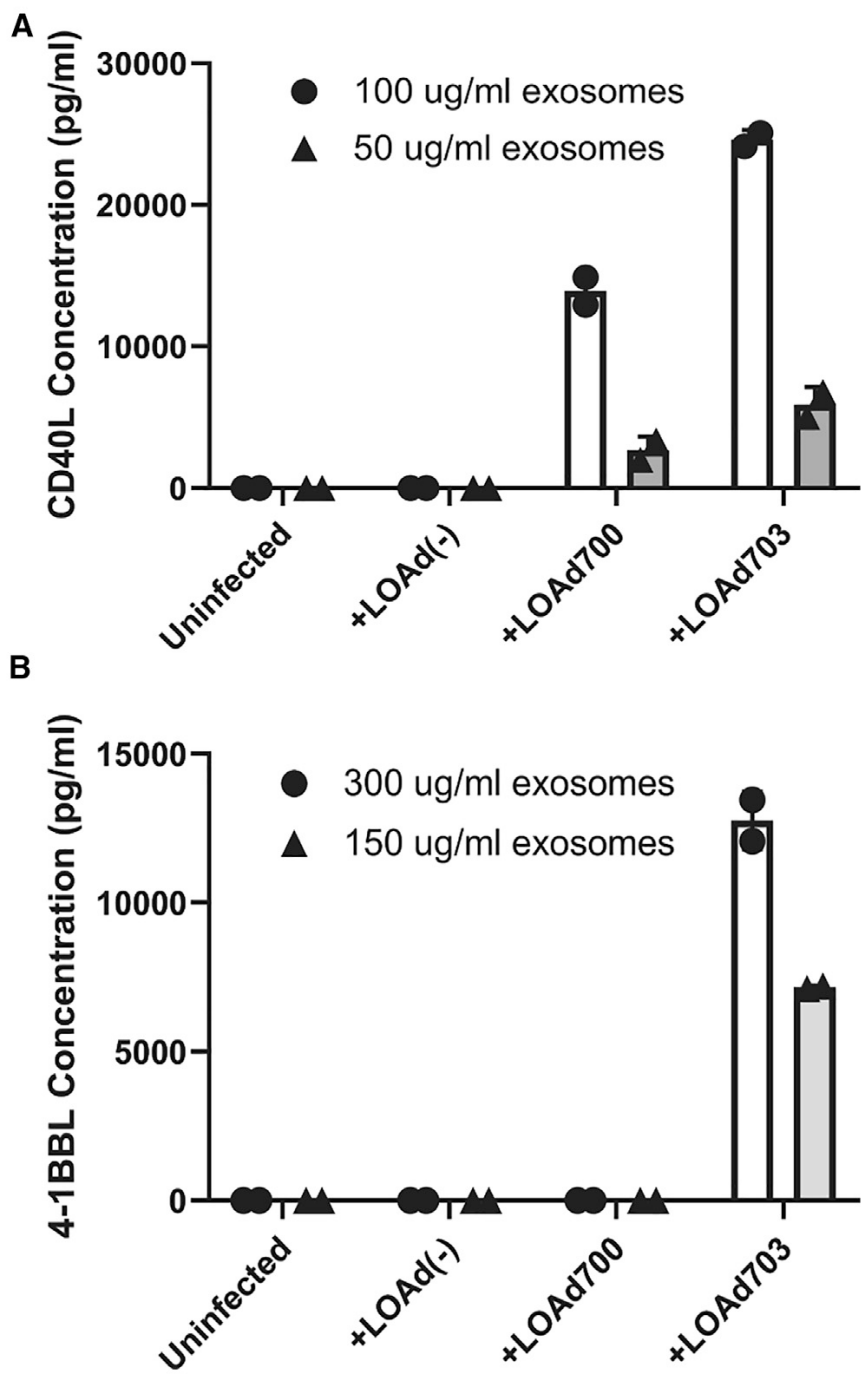
CD40L and 4-1BBL protein content of cells and exosomes (A and B) Exosomes derived from Mel526 cells infected with LOAd viruses or left uninfected were investigated for the presence of CD40L (A) and 4-1BBL (B) with ELISA. Each group was tested in duplicate. Error bars represent standard error and median values are displayed in the graphs.

**Figure 4:**
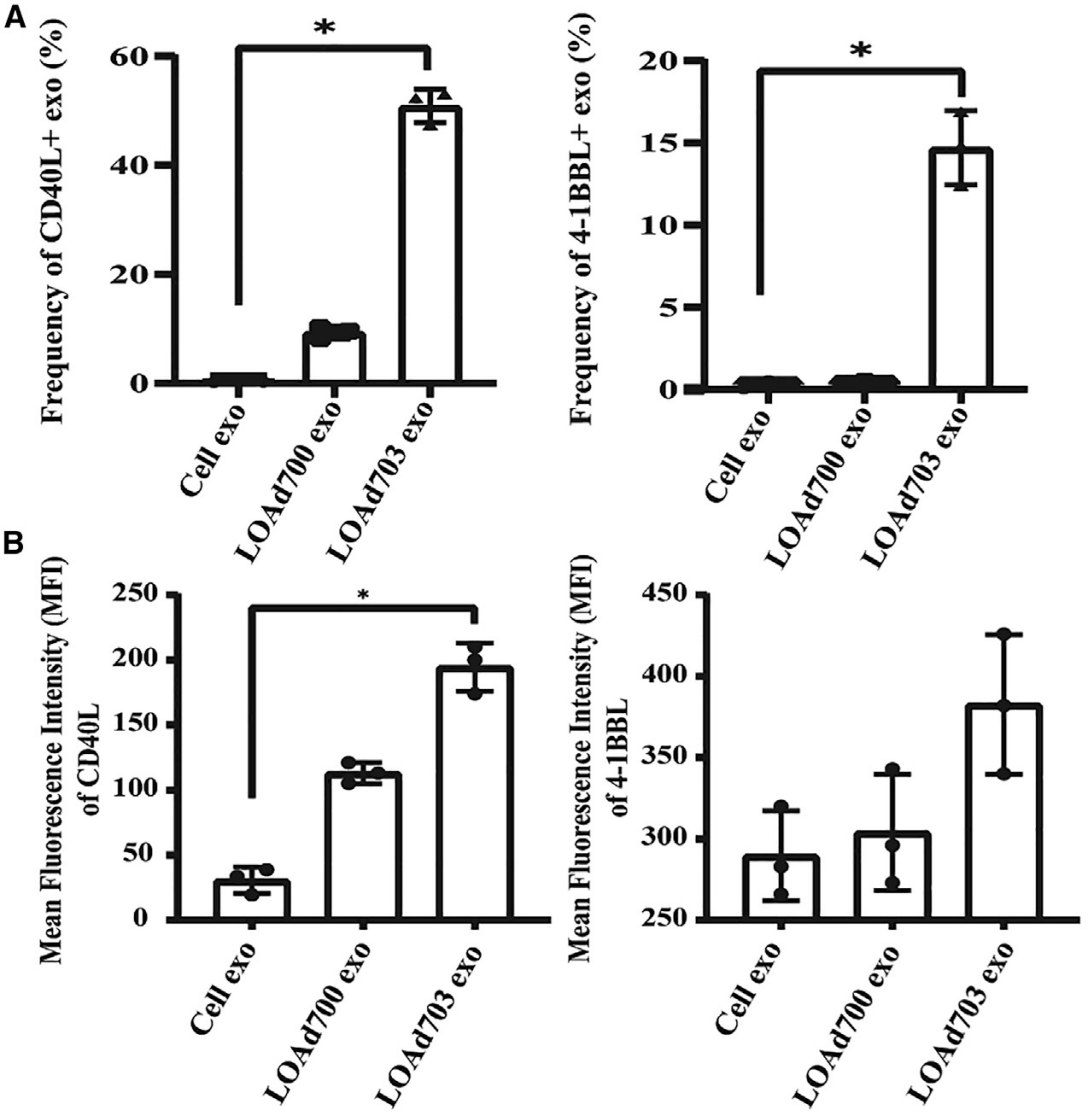
Surface expression of CD40L and 4-1BBL on exosomes from LOAd-infected cells using flow cytometry (A) Total percentage of CD40L^+^ and 4-1BBL^+^ exosomes. (B) Mean fluorescence intensity (MFI) of CD40L and 4-1BBL expression on CD63^+^ bead-coupled exosomes. ∗p ≤ 0.05, n = 3. Error bars represent standard error and median values are displayed in the graphs.

Nanoparticle tracking analysis showed the concentration and a size distribution of about 100 nm of exosomes (Figure 2A). In addition, both exosomal markers, CD63 and CD81, were detected using western blot and enriched on the exosomes (Figure 2B). Negative contrast staining using transmission electron microscopy (TEM) revealed the size, purity, and cup-shaped morphology of exosomes (Figure 2C, upper left). These results show that the isolated vesicles were indeed cell-derived exosomes. To elucidate whether exosomes from the Mel526 cells infected with LOAd700 or LOAd703 cargo the transgenes, different methods were applied. We observed the presence of CD40L on immunogold-coupled exosomes from LOAd700 and LOAd703 using TEM (Figure 2C, upper right and lower left). 4-1BBL expression was seen only on exosomes from LOAd703-infected cells (Figure 2C, lower right). Moreover, we detected CD40L protein in exosomes derived from both LOAd700- and LOAd703-infected cells as well as 4-1BBL protein in exosomes derived from LOAd703-infected cells using ELISA (Figures 3A and 3B). The CD40L and 4-1BBL protein levels were also confirmed with western blot (Figure S2). To reveal the surface expression of the transgenes on cellular exosomes, flow cytometry was applied by gating on CD63^+^ exosomes coupled to beads (Figure S3). Figure 4 shows both the frequency (%) and mean fluorescence intensity (MFI) of transgene-positive exosomes. CD40L was displayed on exosomes from both LOAd700- and LOAd703-infected Mel526 cells. However, exosomes from LOAd703-infected cells had a significantly higher frequency of transgene-positive exosomes compared to controls (p ≤ 0.05) (Figure 4A). The MFI of 4-1BBL was significantly increased in exosomes from LOAd703-infected cells (p ≤ 0.05) (Figure 4B). In addition, both CD40L and 4-1BBL expression was detected on the exosomes isolated from MiaPaCa2, Panc01, and SKOV3 cells infected with LOAd703 but with lower frequencies than that in Mel526 cells (Figure S4).

### Tumor-derived exosomes cargo transgene mRNA after LOAd infection

**Figure 5:**
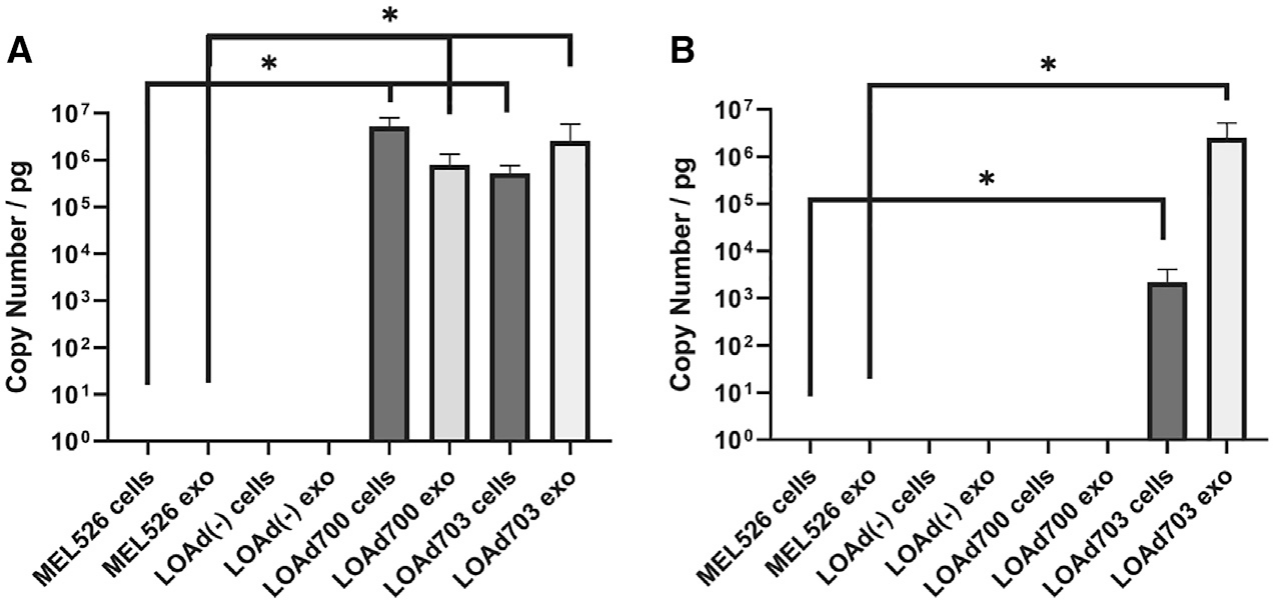
TMZ-CD40L and 4-1BBL mRNA in infected cells and exosomes (copy number per picogram) (A and B) LOAd-infected or uninfected Mel526 cells and cell-derived exosomes were evaluated for their TMZ-CD40L (A) or 4-1BBL (B) mRNA content using quantitative real-time PCR. Samples were analyzed in triplicates (cells) or duplicates (exosomes). Statistical analyses were done using a Kruskal-Wallis test for non-parametric samples with a Dunn’s multi-comparison test. A p value of ≤0.05 was considered significant. n = 3.Error bars represent standard error and median values are displayed in the graphs.

We next investigated whether the exosomes from LOAd-infected cells could cargo transgene mRNA using quantitative reverse-transcriptase PCR (qRT-PCR). CD40L mRNA was significantly increased in exosomes derived from both LOAd700- and LOAd703-infected cells as well as in the infected cells (p ≤ 0.05) (Figure 5A). Neither uninfected or LOAd(−)-infected cells, nor their exosomes, had CD40L mRNA. 4-1BBL mRNA was significantly increased in both LOAd703-infected cells and their exosomes compared to the uninfected cells and exosomes derived thereof (p ≤ 0.05) (Figure 5B).

### LOAd700 and LOAd703 OVs mature and activate DCs *in vitro*

**Figure 6:**
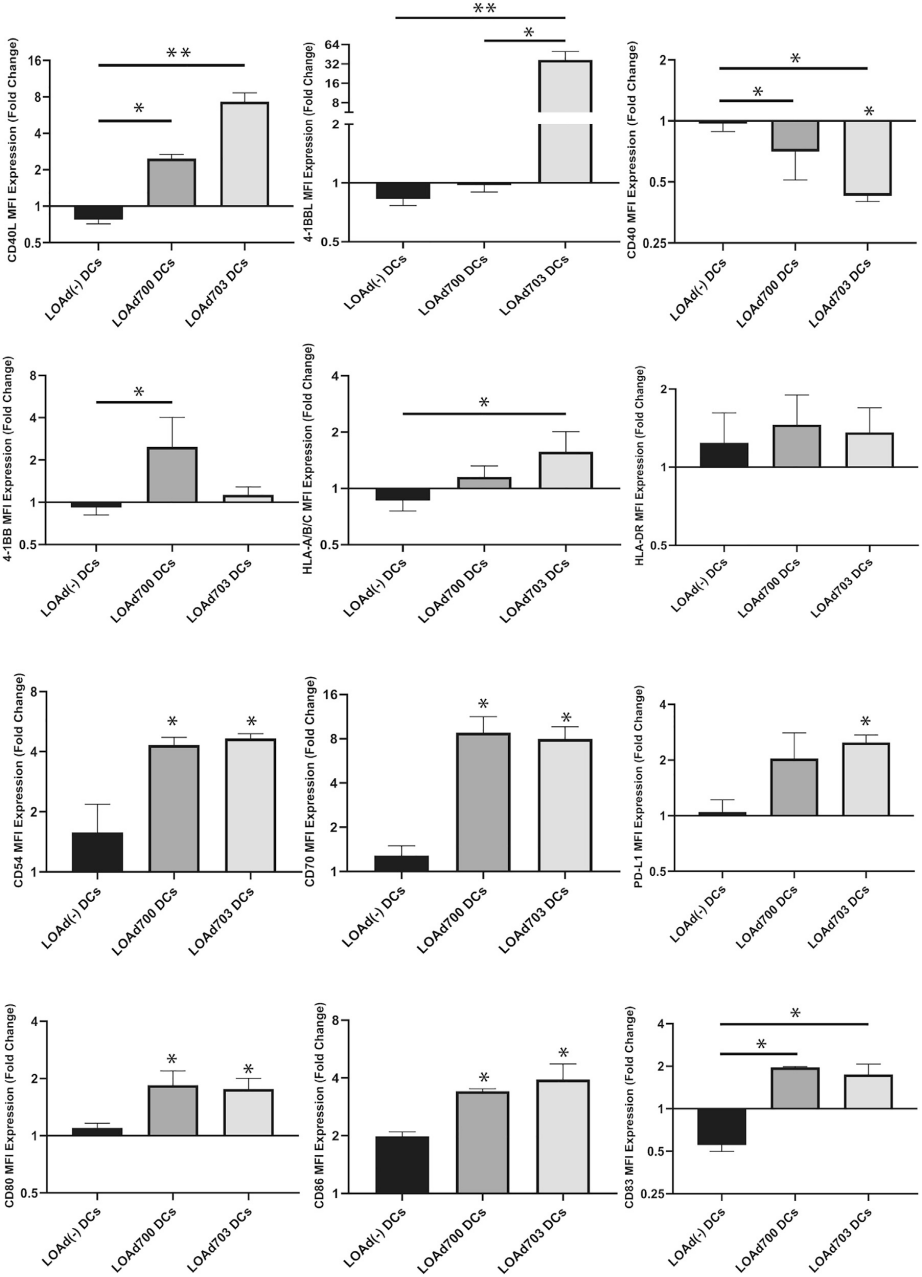
DC activation by LOAd viruses DCs infected by LOAd viruses were analyzed with flow cytometry at day 2 post-infection (n = 3). The fold change of markers expressed by LOAd-infected DCs against uninfected DCs (set as 1) is shown. Asterisks on bars with no straight line are significant changes compared with uninfected DCs. Statistical significances were evaluated using a Kruskal-Wallis test with a Dunn’s multiple comparison test. A p value of <0.05 was considered significant. ∗p ≤ 0.05, ∗∗p ≤ 0.01. LOAd DCs, immature DCs treated with LOAd viruses. Error bars represent standard error and median values are displayed in the graphs.

The LOAd viruses express the transgenes under control of a cytomegalovirus (CMV) promoter, meaning that any infected cell can express the transgenes even if the virus will only replicate in tumor cells. To confirm the ability of the LOAd viruses to stimulate immature DCs *in vitro*, monocytes were isolated from blood samples of healthy individuals (n = 3) and differentiated into immature DCs with granulocyte-macrophage colony-stimulating factor (GM-CSF) and interleukin (IL)-4. The immature DCs were then infected with the LOAd viruses. Both LOAd700 and LOAd703 induced CD40L expression on the CD1a^+^ DCs (p ≤ 0.05 for LOAd700 and p ≤ 0.01 for LOAd703). LOAd703 also induced 4-1BBL expression (p ≤ 0.01) while LOAd(−)-infected DCs did not display the transgenes (Figure S5; Figure 6). The corresponding CD40 and 4-1BB receptors were reduced in DCs expressing the ligand, which is commonly seen due to internalization of the receptor with its ligand. Major histocompatibility complex (MHC) class I (human leukocyte antigen [HLA]-ABC), CD54, CD70, CD80, CD86, and CD83 were all significantly increased by both LOAd700- and LOAd703-infected DCs (p ≤ 0.05) (Figure 6). Upon activation, DCs upregulate PD-L1 and, in this experiment, PD-L1 was significantly upregulated in LOAd703-infected DCs (p ≤ 0.05).

### Exosomes armed with both TMZ-CD40L and 4-1BBL activate DCs

**Figure 7:**
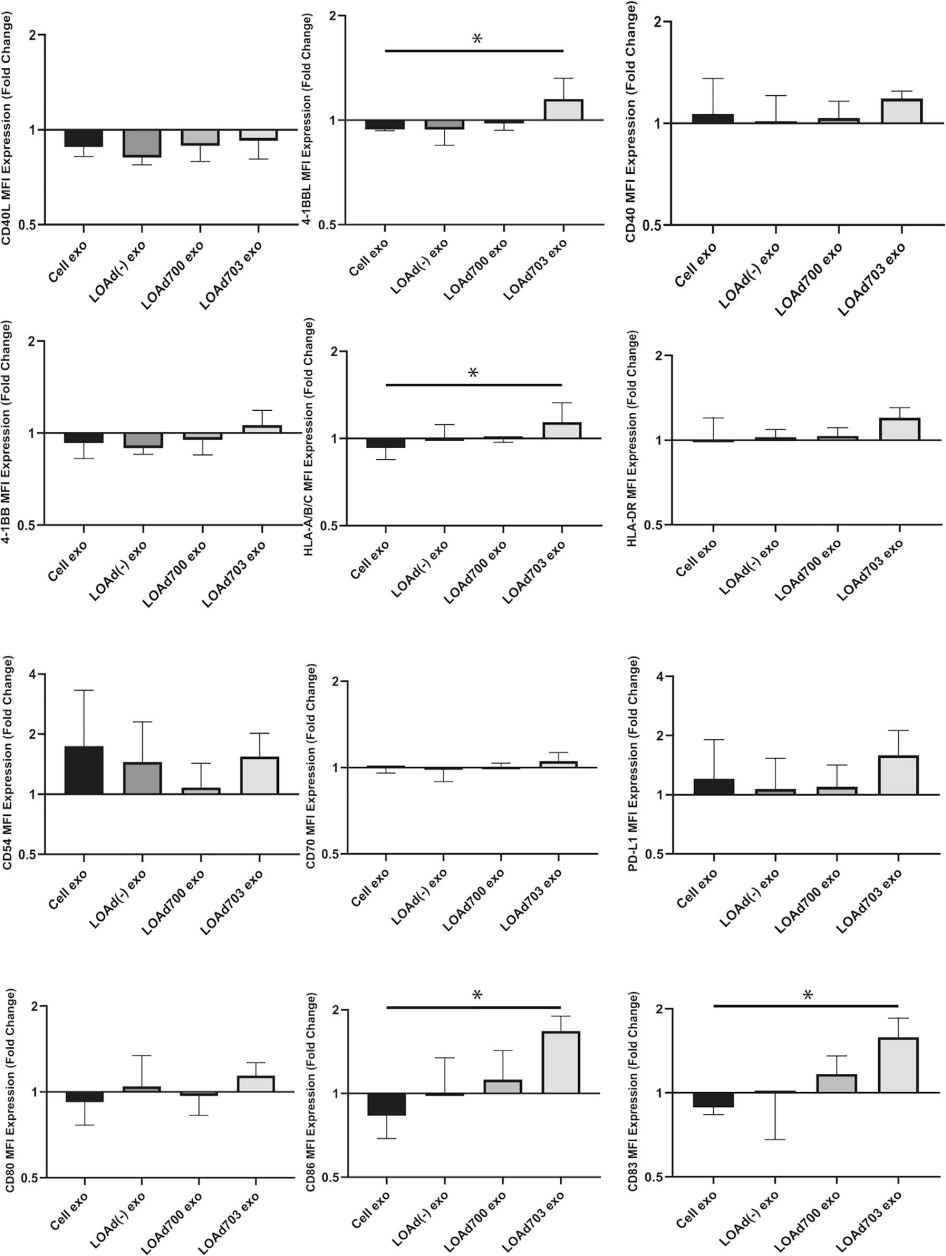
DC activation by exosomes Exosomes derived from Mel526 cells infected with LOAd viruses were used to stimulate DCs. DCs were analyzed at day 2 post-stimulation with flow cytometry (n = 3). Fold change markers after stimulation by exosomes against unstimulated DCs are shown. Statistical significance was evaluated using a Kruskal-Wallis test with a Dunn’s multiple comparison test. A p value of <0.05 was considered significant. ∗p ≤ 0.05. Error bars represent standard error and median values are displayed in the graphs.

Exosomes derived from tumor cells are commonly immunosuppressive.^15^, ^16^, ^17^ In line with this, addition of exosomes derived from uninfected tumor cells to immature DCs decreased antigen presentation and costimulation, while PD-L1 was upregulated. Since both LOAd700 and LOAd703 can activate immature DCs, we hypothesized that exosomes that cargo the same transgenes (TMZ-CD40L and 4-1BBL) can instead activate immature DCs. For this purpose, immature DCs were treated with exosomes derived from Mel526 cells infected with LOAd700, LOAd703, or LOAd(−), or left uninfected. Stimulation using 20 μg of exosomes every second day did not activate the DCs (Figure S6). In the next experiment, the dose was increased to 40 μg every second day, which may better correspond to levels released from a growing tumor, as 40 μg of exosomes consists of almost 1.14 × 10^10^ particles released by approximately 2 × 10^7^ melanoma cells that correspond to about 0.2 g of tumor mass (0.2 cm^3^).^18^ Strikingly, the higher dose of exosomes derived from LOAd703-infected tumor cells could mature DCs, as seen by upregulation of MHC class I (HLA-ABC), CD86, and CD83 (p ≤ 0.05) (Figure 7). Other markers such as PD-L1, MHC class II (HLA-DR), CD40, CD40L, CD54, 4-1BB, and CD70 were affected, but the difference was not significant using nonparametric tests (Figure 7). Nevertheless, 4-1BBL (p ≤ 0.05) was significantly upregulated by exosomes derived from LOAd703-infected cells (Figure 7). To confirm that there was no LOAd703 virus contaminating the purified exosomes that could be the agent stimulating the DCs, we re-examined the TEM images and could not see any contaminating adenovirus particles. Furthermore, purified exosomes were added to Panc01 cells, which are very sensitive to LOAd virus-mediated oncolysis, and a viability MTS (3-(4,5-dimethylthiazol-2-yl)-5-(3-carboxymethoxyphenyl)-2-(4-sulfophenyl)-2*H*-tetrazolium) assay was performed. Supernatants from uninfected and infected cells were used as negative and positive controls of virus-mediated oncolysis. The data clearly demonstrate that exosomes from uninfected or LOAd703-infected Mel526 cells could not induce oncolysis of Panc01 cells, while supernatant from infected cells, or purified virus, could potently induce oncolysis (Figure S7). Hence, the purified exosome preparation from infected cells does not contain contaminating viral particles.

## Discussion

The immune system plays a pivotal role in cancer. Clinical outcomes depend on the level of TILs in the TME, as an augmentation of CD4^+^, CD8^+^, and NK cells in the early stage of a malignant tumor is associated with a favorable prognosis. In contrast, the enhancement of tumor-associated stroma cells, inhibitory receptors, suppressive metabolites, and soluble mediators leads to immune suppression and is correlated with poor prognosis.^2^ Cancer immunotherapy aims to activate or restore antitumor immune responses. The use of antibodies that block inhibitory checkpoint signaling in T cells has revolutionized cancer therapy.^19^ Nevertheless, such a treatment depends on pre-existing immune reactive TILs, and for non-responsive or refractory tumors there is a need to develop immunotherapies that activate *de novo* immune responses as well as boosting pre-existing immunity to sensitize “cold” tumors to CPIs. Oncolytic virotherapy has demonstrated the capacities of tumor eradication, immune activation, and attracting T cells to the tumor bed.^20^ Nevertheless, due to the immunogenicity of viruses, they are commonly administered by intratumoral injection, and it has been debated whether local administration can result in systemic immunity. In this study, we demonstrate a novel mechanism of action utilized by OVs to arm tumor-derived exosomes with their immunostimulatory transgenes, and this may partially explain why local treatment can result in systemic immunity.

Tumor-derived exosomes are known to support immunosuppression. For example, the immunosuppressive function of melanoma-derived exosomes has been demonstrated in several studies. It was shown that melanoma exosomes expressing PD-L1 suppress CD8^+^ T cells.^15^ Furthermore, melanoma exosomes promote M1 and M2 macrophage polarization via upregulation of cytokines IL-10 and TNF-α.^21^ Moreover, tumor-derived exosomes in plasma of melanoma patients inhibited proliferation and function of effector T cells and down-modulated the expression of NKG2D in NK cells.^22^ The anti-immunity function of melanoma exosomes was also due to microRNA (miRNA) transcripts, as demonstrated in a few studies.^23^^,^^24^ Valenti et al.^25^ added melanoma-derived microvesicles to CD14^+^ monocytes and observed that the cells retained CD14 status, had HLA-DR (−/low) expression, could not upregulate co-stimulatory molecules, and exhibited an inhibitory function via transforming growth factor (TGF)-β against T cells. Moreover, they were highly expanded in the peripheral blood of patients with melanoma. Ning et al.^17^ used exosomes from other cancer cell lines than melanoma and indicated that they suppressed the maturation and migration of murine bone marrow-derived DCs, which were partially due to PD-L1^+^ exosomes. Nevertheless, we hypothesized that the overexpression of immunostimulatory molecules in tumor cells by OVs may counteract the suppressive function of exosomes. We have developed an oncolytic adenovirus platform (LOAd) armed with immunostimulatory transgenes such as TMZ-CD40L and 4-1BBL that target DCs as well as T and NK cells.

LOAd viruses were previously shown to kill tumor cells by oncolysis, induce the expression of immunostimulatory transgenes in tumor cells, DCs, and stroma, to activate APCs such as DCs, and to expand antigen-specific T and NK cells.^6^^,^^7^ In this study, we used an *in vitro* model of melanoma (Mel526). Mel526 cells were infected with LOAd viruses such as LOAd700 and LOAd703, and thereafter a robust expression of 4-1BBL and/or TMZ-CD40L was noted on the cell surface. Isolated exosomes from infected Mel526 cells showed all anticipated features such as cup-shaped form, size, and the exosomal markers CD63 and CD81. Sorting and loading molecules in the endosomal compartments during exosome biogenesis are a selective process, but 4-1BBL and TMZ-CD40L were both displayed on the exosome as surface protein and loaded as mRNA in the exosome core. As these two molecules are potent stimulators of T helper (Th)1 type immunity, such exosomes have the potential to stimulate immune activation.

In cancer, DCs are commonly immature due to the suppressive TME. Since immature DCs lack or have low expression of co-stimulatory molecules and a low level of MHC molecules, they cannot induce T cell responses.^26^ It was demonstrated that 4-1BB is expressed by activated DCs,^27^ and secretion of IL-12 is a result of 4-1BB stimulation on DCs.^28^ This cytokine consequently promotes a Th1 response and elicits IFN-γ secretion by NK and T cells.^29^ Activation of CD40 on DCs leads to induction of cytokine production and costimulatory molecules through several signaling pathways.^11^ Both LOAd700, armed with TMZ-CD40L, and LOAd703, armed with TMZ-CD40L as well as 4-1BBL, could directly stimulate DCs post- infection since the viruses induced transgene expression in the DCs. Both LOAd700 and LOAd703 upregulated MHC molecules (HLA-A/B/C and HLA-DR), costimulatory molecules (CD70, CD80, and CD86), adhesion molecules (CD54), and the CD83 activation marker that are all important in DC and T cell interactions. However, PD-L1 was also upregulated, which is a consequence of DC maturation. In contrast, LOAd(−) that lacks immunostimulatory transgenes was not a potent DC stimulator. After confirming DC maturation by TMZ-CD40L and 4-1BBL by direct gene transfer, we treated immature DCs every 2 days with exosomes isolated from melanoma cells infected with either LOAd viruses or left uninfected. Exosomes derived from LOAd703-infected Mel526 cells could significantly enhance the maturation of DCs. The maturation was not as robust as seen with direct infection of LOAd viruses, but since the viruses will induce overexpression of the transgenes, the concentration of each transgene will be much higher. Exosomes derived from LOAd700-infected Mel526 cells did not significantly upregulate DC maturation markers, but both CD86 and CD83 tended to be increased compared to DCs stimulated with exosomes from LOAd(−)-infected or uninfected Mel526 cells. Of interest, exosomes from uninfected Mel526 cells tended to decrease the maturation marker while PD-L1 tended to increase it, which are in line with previous data.^15^^,^^17^^,^^21^, ^22^, ^23^, ^24^, ^25^

Viruses have been shown to hijack microvesicles to spread and evade immune recognition.^14^ Microvesicles including exosomes could cargo viral particles and components.^30^^,^^31^ For example, latent membrane protein 1 (LMP1) from Epstein-Barr virus (EBV) can be sorted into exosomes and released by virus-infected cancer cells to eliminate the activation and proliferation of tumor-infiltrating T cells.^30^ However, the members of Adenoviridae have a size about 90 nm, whereas exosomes are between 30 and 150 nm.^12^^,^^32^ Because of their similar size, it does not seem feasible for exosomes to transfer adenoviral particles but their components such as proteins and genomic materials. Therefore, DC activation by exosomes from LOAd703-infected melanoma cells was not because of transfer of viral particles but rather the transgenes as mRNA and proteins. As exosomes from LOAd(−)-infected cells did not stimulate DC maturation, viral components possibly packed into exosomes were likely not contributing to DC maturation. Moreover, we conducted a relative viability test to determine whether the LOAd703 exosomal preparation was contaminated with LOAd703 virus and, hence, cells mixed with the exosomal preparation would die by oncolysis. We could not detect any oncolysis of the LOAd703-sensitive cell line Panc01 cells upon addition of exosome preparations from infected or uninfected cells. Furthermore, TEM did not reveal any contaminating virus particles.

Taken together, LOAd-infected melanoma cells released exosomes that cargo the transgenes encoded by the viruses. Exosomes from LOAd703-infected melanoma cells could mature DCs in a dose-dependent manner. Hence, OVs based on adenoviruses can distribute their immunostimulatory transgenes via tumor-derived exosomes and activate DCs that encounter the stimulatory exosomes. As exosomes travel via vessels, DCs at distant sites may also be activated, but further studies are needed to confirm the long distance ability of exosome-mediated immune activation *in vivo*.

## Materials and methods

### Cell line

The melanoma cell line Mel526 was a gift from Prof. Magnus Essand (Uppsala University, Uppsala, Sweden) and was cultured in RPMI 1640 supplemented with l-glutamine, 10% fetal calf serum (FCS), and 1% penicillin/streptomycin (PeSt). Two human pancreatic cell lines (MiaPaCa2 and Panc01) were gifts from Dr. Rainer Heuchel (Karolinska Institute, Stockholm, Sweden) and cultured in DMEM supplemented with 10% FCS and 1% penicillin/streptomycin. The ovarian cancer cell line SKOV3 was purchased from ATCC (Manassas, VA, USA) and cultured in McCoy’s medium supplemented with 10% FCS and 1% penicillin/streptomycin. All media and supplements were purchased from Thermo Fisher Scientific (Waltham, MA, USA). The cells were incubated at 37°C in 5% CO_2_ and humidity. To investigate the surface expression of CD40L and 4-1BBL, Mel526 cells were infected with an MOI of 10 fluorescence-forming units (FFU)/cell of LOAd703, LOAd700, or LOAd(−) for 2 h and incubated for 48 h at 37°C prior to cell harvest and staining for flow cytometry.

### LOAd viruses

LOAd viruses were constructed as described previously and were provided by Lokon Pharma AB (Uppsala, Sweden).^6^^,^^7^ Briefly, the gene sequence of TMZ-CD40L (LOAd700) or along with 4-1BBL (LOAd703) was synthesized and placed under control of a CMV promoter before the right inverted terminal repeats (RITRs) at the 3′ end of the LOAd genome. Viruses were produced via LOAd genome plasmid transfection of 293 cells, expression of the virus supernatants in A549 cells, and purified by CsCl gradient centrifugation prior to formulation and titration. LOAd(−) was used as a backbone adenovirus with no transgene cassette.

### Isolation of exosomes

Mel526 cells were infected with an MOI of 10 FFU/cell of LOAd(−), LOAd700, or LOAd703 using serum-free medium and incubated for 2 h at 37°C. When the cells were about 80% confluent, they were washed twice with PBS before medium containing ultracentrifuged FCS (100,000 × *g* for 16 h) was added into the culture flasks and incubated at 37°C for 48 h prior to the exosome isolation procedure. To isolate exosomes, the cell medium was spun at 250 × *g* for 5 min, 2,500 × *g* for 30 min, and at 10,000 × *g* for 30 min at 4°C (Beckman Avanti J-25). The supernatant was passed through a 0.22-μm filter before ultracentrifugation. The supernatant was spun at 110,000 × *g*, 4°C for 2 h using rotor AH 629 (Sorvall WX Ultra 80, Thermo Scientific). The resuspended pellet was washed with filtered PBS at 110,000 × *g*, 4°C for 2 h (Beckman Coulter). The exosomal pellet was finally resuspended with filtered PBS and Pefabloc SC (AEBSF [4-(2-aminoethyl)benzenesulfonyl fluoride hydrochloride]) as a protease inhibitor (Roche, Mannheim, Germany). The protein concentration of the isolated exosomes was determined with bicinchoninic acid (BCA) (Pierce) at absorbance of 562 nm using a NanoDrop 1000 (Thermo Scientific). They were stored at −80°C until use. The isolation of exosomes from MiaPaCa2, Panc01, and SKOV3 cells infected with LOAd703 was the same as described for Mel526.

### Nanoparticle tracking analysis

Based on Brownian movement, the size distribution and number of the isolated exosomes were characterized using NanoSight NS500 (Malvern Panalytical, UK). Exosomes were diluted with particle-free PBS prior to their collection by the instrument. The average concentration was calculated from five captured videos.

### TEM

Negative contrast staining was used to evaluate the morphology and purity of the exosomes. Immunoelectron microscopy (IEM) was performed to reveal the expression of TMZ-CD40L and 4-1BBL on exosomes. Briefly, exosomes were fixed with 2% paraformaldehyde (PFA) and adsorbed to gold formvar/carbon-coated grids (Ted Pella). Exosomes were then incubated with a primary antibody, either CD40L (AF617, R&D Systems) or 4-1BBL (ab126274, Abcam), followed with goat anti-mouse or goat anti-rabbit as a secondary antibody conjugated with 6-nm gold particles (Jackson ImmunoResearch) in a ratio of 1:80 (40 min incubation each). Finally, exosomes were observed with an electron microscope (Tecnai Biotwin) at 80 kV. For negative contrast staining, exosomes were treated with 1% glutaraldehyde and a drop of uranyl-oxalate solution, followed with 4% uranyl acetate and 2% methylcellulose after fixation and adsorption to the grids.

### Western blot

Equal concentration of exosomes and cell lysates were loaded in mini-protean TGX precast gel (Bio-Rad) after addition of sample buffer and a heating step. The proteins were then transferred to polyvinylidene fluoride (PVDF) membranes (GE Healthcare). Blocking of the membranes was performed utilizing 3% ECL prime blocking agent (Amersham, GE Healthcare) for 1 h at room temperature. The membranes were exposed to an appropriate amount of primary antibodies such as CD40L (AF617, R&D Systems), 4-1BBL (ab126274, Abcam), GAPDH (mAbcam 9484, Abcam), CD63 (MX-49.129.5, Santa Cruz), or CD81 (5A6, Santa Cruz) and incubated at 4°C overnight while rotating. After a washing step, peroxidase-conjugated secondary antibodies such as mouse-immunoglobulin Gκ (IgGκ; BP-HRP, Santa Cruz), horseradish peroxidase (HRP)-conjugated anti-goat IgG (HAF109, R&S Systems), or HRP-conjugated anti-rabbit IgG (ab97051, Abcam) were added to the membranes, which were then washed with PBS/0.05% Tween 20 before development by enhanced chemiluminescence (ECL) prime western blotting detection reagents (Amersham, GE Healthcare). The protein bands were visualized by a ChemiDoc touch imaging system (Bio-Rad).

### qRT-PCR

Total RNA was extracted from Mel526 cells that were either uninfected or infected with LOAd viruses (10 FFU/cell) 48 h after infection or from the exosomal pellets isolated from uninfected or LOAd-virus-infected Mel526 cells using an RNeasy micro kit (QIAGEN). The total RNA samples were eluted with 14 μL of RNase-free water and kept at −80°C until further use. The total RNA samples were DNase treated (Promega) prior to cDNA synthesis. The cDNA of the samples was synthesized using iScript reverse transcription supermix with a PTC-200 Peltier thermal cycler. The cDNA of the exosomes was not diluted, while the cDNA of the cells was diluted 1:100. Quantitative PCR was performed using SYBR Green and a CFX96 touch real-time PCR detection system (Bio-Rad). The cDNA samples of the cells and exosomes were run as triplicates and duplicates, respectively. Duplicates of non-RT (NRT) and different dilutions of standards of both TMZ-CD40L and 4-1BBL plasmid were included along with non-template control and 50 pg/μL plasmid containing both transgenes as positive controls. The samples were run for 40 cycles. Using the standard curve, the copy numbers were calculated.

### ELISA

CD40L and 4-1BBL were detected in exosomes isolated from Mel526 cells infected with LOAd700 or LOAd703 viruses by ELISA. ELISA was performed using a human CD40L ELISA kit (ELH-CD40L) and a human 4-1BBL ELISA kit (ELH-41BBL) (RayBiotech). For CD40L, 100 and 50 μg/mL exosomes, and for 4-1BBL, 300 and 150 μg/mL exosomes were utilized, each in duplicates. Two different exosome concentrations were used to determine the value range of detection for protein concentrations. The plates were read with an iMark microplate reader (Bio-Rad).

### DC stimulation assay

Peripheral blood mononuclear cells (PBMCs) were isolated from buffy coats, obtained from three healthy donors under a reserach blood bank ethical permit (Blood Bank at Uppsala University Hospital, Uppsala, Sweden), using Ficoll-Paque gradient centrifugation. CD14^+^ monocytes were then separated from the PBMCs using human CD14^+^ magnetic beads (Miltenyi Biotec). The monocytes were differentiated into immature DCs by culturing them in RPMI 1640 supplemented with 10% FCS, 1% HEPES, 1% PeSt, and 0.2% β-mercaptoethanol, all purchased from Thermo Fisher Scientific (Waltham, MA, USA), as well as GM-CSF (150 ng/mL) and IL-4 (50 ng/mL) (both cytokines from PeproTech) for 6 days. 10^6^ immature DCs were either stimulated with 40 μg of purified exosomes (LOAd-700, LOAd-703, LOAd(−), or untreated Mel526 cells) every 2 days, or infected with 10 FFU/cell of LOAd viruses (LOAd-700, LOAd-703, or LOAd(−)) or left uninfected. After 48 h, cells were harvested and stained with antibodies, washed, and spun and the cell pellets were resuspended and fixed in PBS containing 1% PFA and 3 mM EDTA before analysis with BD FACSCanto II (BD Biosciences, San Jose, CA, USA). The antibodies utilized for staining were anti-human CD1a (HI149) from BD Biosciences (Franklin Lakes, NJ, USA) and CD63 (H5C6), CD14 (HCD14), CD40L (24-31), 4-1BBL (5F4), CD40 (HB14), 41BB (4B4-1), CD80 (2D10), CD83 (HB15e), CD86 (IT2.2), CD70 (113-16), CD54 (HCD54), CCR7 (G043H7), HLA-DR (L243), HLA-A/B/C (W6/32), PD-L1 (29E2A3), CD16 (3G8), IgG1 (MOPC-21), IgG2a (MOPC-173) and IgG2b (MPC-11), all from BioLegend (San Diego, CA, USA). Data were assessed in FlowJo (Tree Star, Ashland, OR, USA). Dead cells and duplets were removed by forward and side scatter gating.

### Exosome preparation for flow cytometry or functional DC studies

For performing flow cytometry of the cellular exosomes, 5 μL of nanovesicles isolated with ultracentrifuge was coupled to 20 μL of CD63^+^ beads using exosome-human CD63 isolation/detection (Invitrogen) for 18–22 h according to the manufacturer’s instruction. The bound exosomes were then stained with CD63 (H5C6), 4-1BBL (5F4), CD40L (24-31), and mouse IgG1 as isotype control, all purchased from BioLegend (San Diego, CA, USA), with incubation at room temperature for 60 min in the dark while shaking at 1,000 rpm. Then, the exosomes were washed twice and separated with a magnet for 1 min before starting flow cytometry analysis. The gating was based on the CD63^+^ exosome population. For functional DC studies, exosomes were coupled to CD63^+^ magnetic beads at 4°C overnight. After incubation, the bead-bound exosomes were washed and then treated with 50 mM glycine for 2 min at room temperature three times to dissociate the CD63^+^ exosomes from the beads.

### MTS assay

To perform the MTS assay, 1 × 10^6^ Panc01 cells were infected with 10 FFU/cell LOAd703, incubated at 37°C for 2 h, and then 1 × 10^4^ cells were plated in triplicates. The same cell number was left uninfected either alone or co-cultured with exosomes/cell culture supernatants as follows: Panc01 cells were co-cultured with exosomes isolated from Mel526 or LOAd703-infected Mel526 cells, and the exosomes had equal concentrations; Panc01 cells were co-cultured with the supernatant of previous exosome isolation from LOAd703-infected Mel526 cells, filtered, ultracentrifuged, and stored at −80°C; or Panc01 cells were co-cultured with fresh supernatant of Panc01 either infected with LOAd703 or left uninfected and incubated for 72 h prior to the test. All procedures were performed in triplicate. The plate was incubated at 37°C for 72 h. The viability of the cells was evaluated with CellTiter 96 AQueous One solution MTS reagent (Promega, Fitchburg, WI, USA).

### Statistical analysis

Statistical analysis of the data was performed using GraphPad Prism 8 (GraphPad, San Diego, CA, USA). For nonparametric data, a Kruskal-Wallis test with a Dunn’s multiple comparison test was used for analysis. p values of ≤0.05 were regarded as significant.

## Supplemental information

Document S1. Figures S1–S7

Document S2. Article plus supplemental information
