# Supplementary material for: Systemic immunity upon local oncolytic virotherapy armed with immunostimulatory genes may be supported by tumor-derived exosomes
Source: Mol Ther Oncolytics. 2021 Feb 17;20:508–18. doi: 10.1016/j.omto.2021.02.007 (PMC7940707; doi:10.1016/j.omto.2021.02.007)
Supplement: Document S1. Figures S1–S7 [file mmc1.pdf]

OMTO, Volume *20*

Supplemental information

Systemic immunity upon local oncolytic virotherapy armed with immunostimulatory genes may be supported by tumor-derived exosomes

Alireza Labani-Motlagh, Sedigheh Naseri, Jessica Wenthe, Emma Eriksson, and Angelica Loskog

Figure S1.

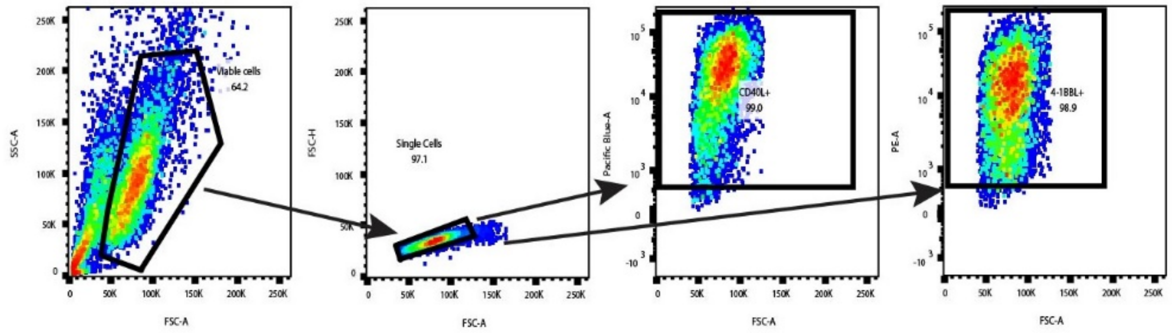

Figure S1. Gating strategy for evaluating transgene expression on melanoma cells. Viable Mel626 cells were gated based on size using a side and forward scatter plot. Singlets were selected and used to evaluate the expression of CD40L and 4-1BBL.

Figure S2.

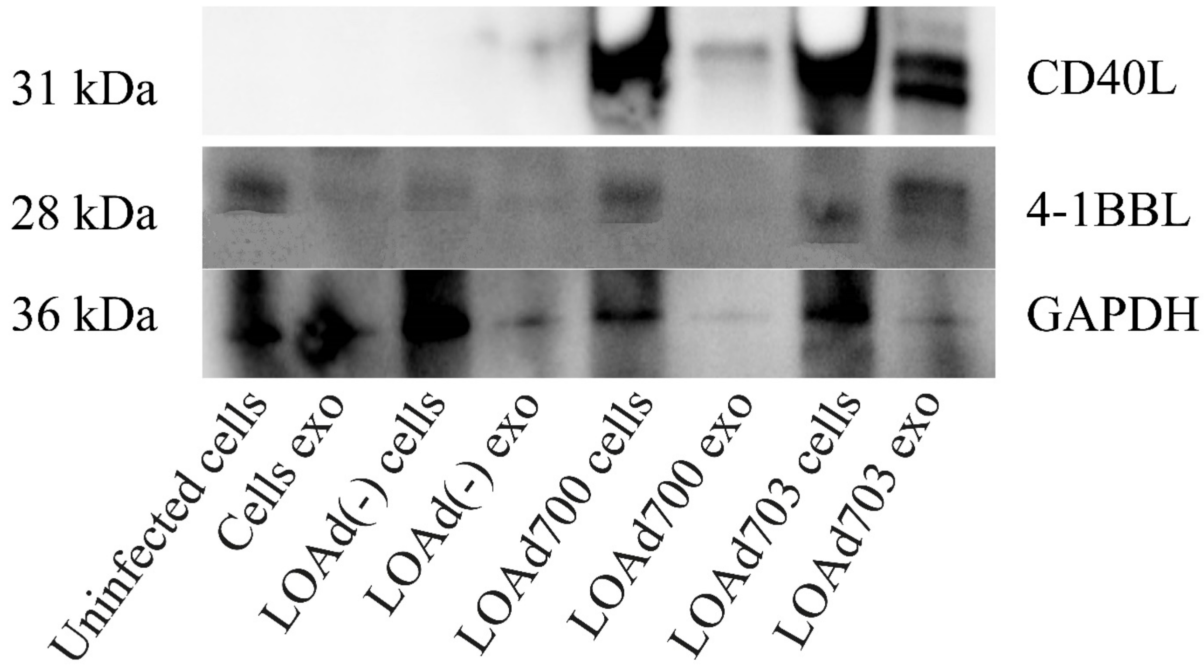

Figure S2. Protein expression of the transgenes detected with Western blot. Mel526 cells were infected with LOAd viruses or left uninfected. At day 2, cells were harvested, lyzed and the cell lysates together with exosomes were evaluated by Western blot. Upper row: CD40L (31 kDa) and middle row: 4-1BBL (28 kDa). GAPDH (36 kDa) was used as a loading control (lower row). LOAd cells = the lysate of cells infected with LOAd viruses. LOAd exo = the lysate of exosomes released by LOAd-infected cells.

Figure S3.

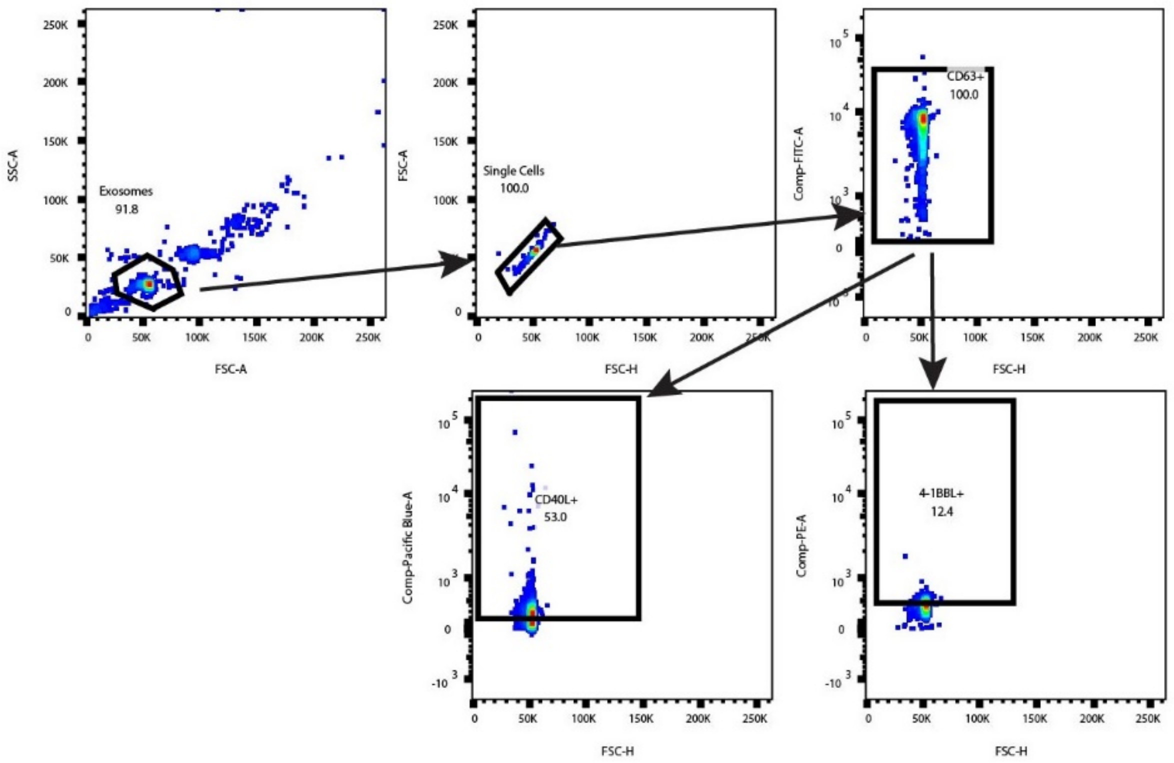

Figure S3. Gating strategy transgene analysis on exosomes. Exosomes were gated based on size using a forward and side scatter plot followed by selection of singlets. Exosomes were then selected based on CD63 expression. Finally, the CD63+ exosomes were used to determine the expression of CD40L or 4-1BBL.

Figure S4.

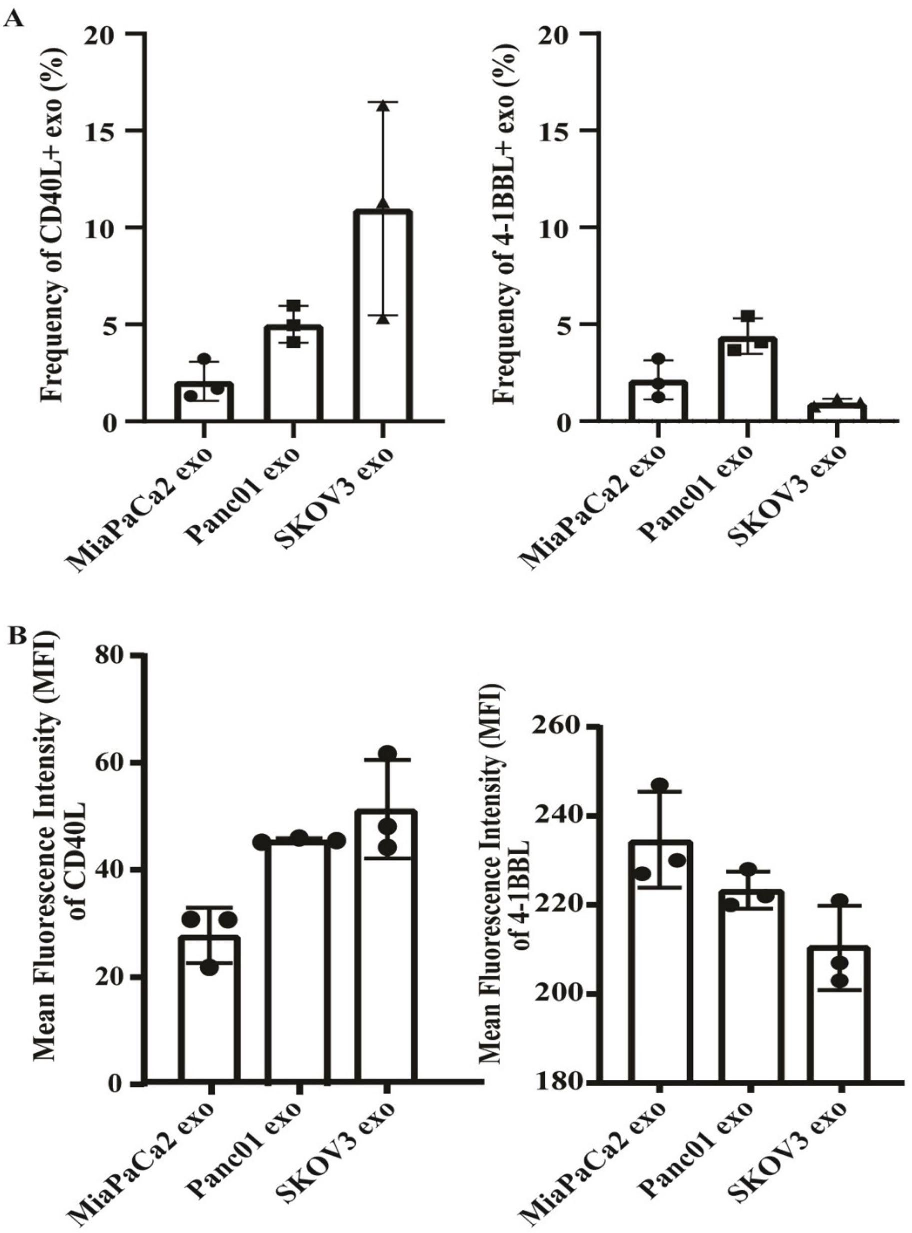

Figure S4. Frequency (A) and mean fluorescence intensity (B) of CD40L- and 4-1BBL-positive exosomes from three cell lines MiaPaCa2, Panc01 and SKOV3 infected with LOAd703 analyzed with flow cytometry (n = 3).

Figure S5.

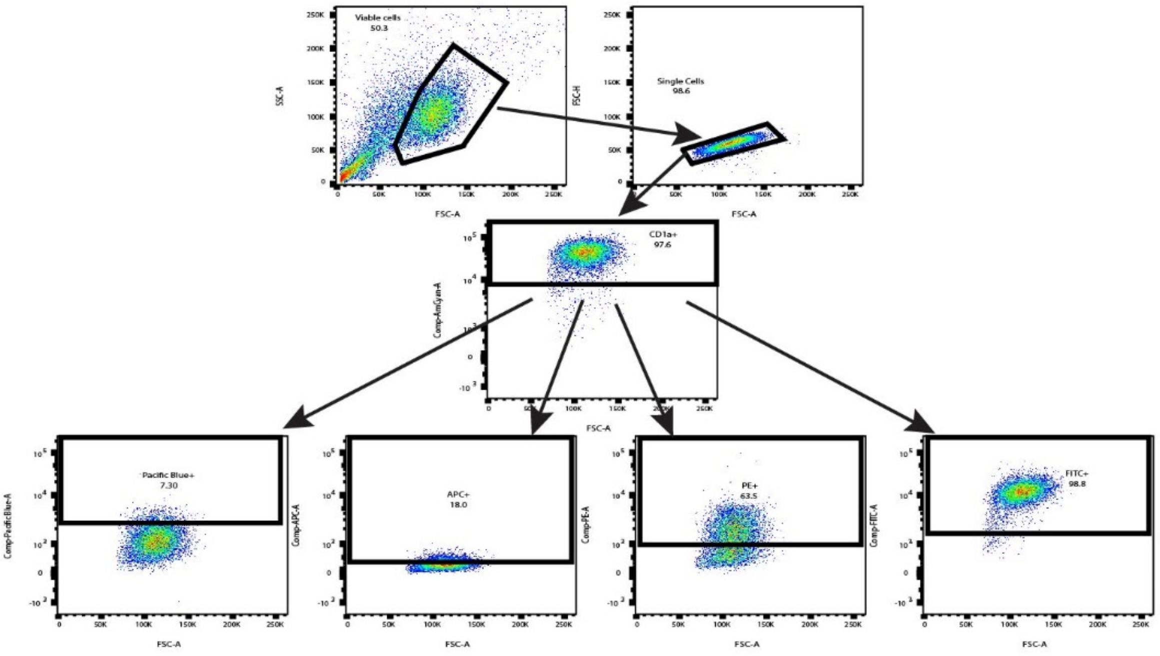

Figure S5. Gating strategy for DC marker evaluation. Live cells were gated using both forward and side scatter plot followed by selection of singlets. CD1a+ DCs were gated prior to selection of cell populations positive for FITC, APC, PE and Pacific Blue.

Figure S6.

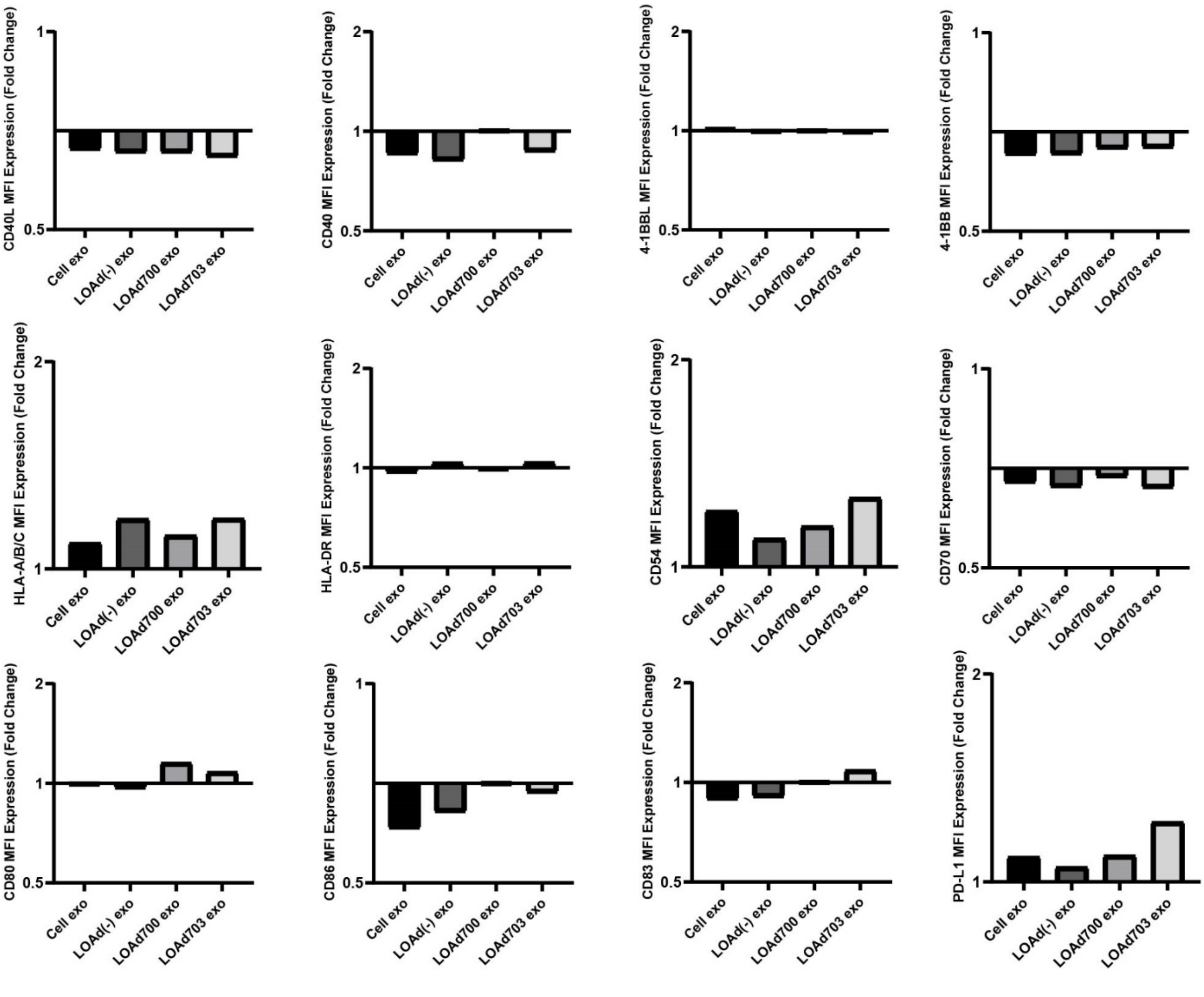

Figure S6. Treatment of immature DCs with 20 μg of exosomes derived from Mel526 cells either infected with LOAd viruses or left uninfected. The cells were harvested, stained and analyzed with flow cytometry at day 2 post treatment (n = 1). Fold change of markers expressed by DCs is shown against uninfected cells. LOAd exo = exosomes from LOAd-infected cells.

Figure S7.

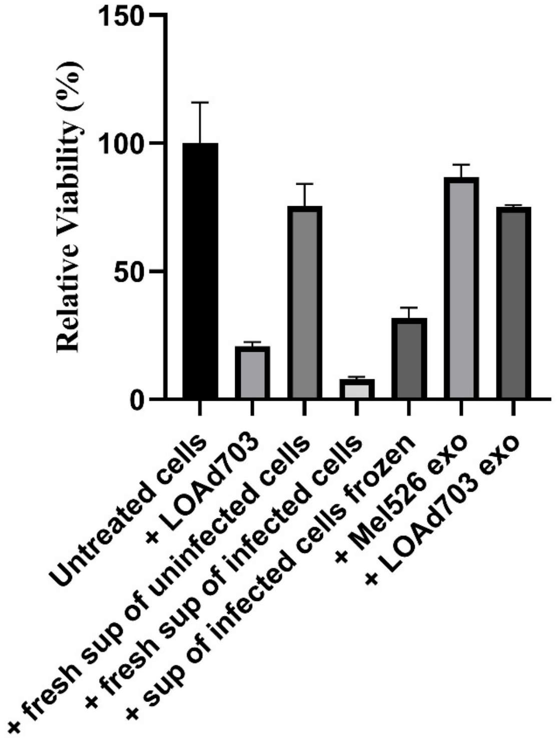

Figure S7. MTS assay. Relative viability (%) of Panc01 cells either untreated or treated with LOAd703, fresh supernatant of uninfected Panc01 cells, fresh supernatant of the Panc01 cells infected with LOAd703, supernatant of LOAd703-infected Panc01 cells after pelleting exosomes and stored at -80°C, exosomes from uninfected Mel526 stored at -80°C, and exosomes from LOAd703-infected Mel526 stored at -80°C.
